# Sleep Quality, Psychological Inflexibility, and Work–Private-Life Interference in Relation to Emotional Exhaustion Among Ecuadorian Nursing Assistants: A Cross-Sectional Study

**DOI:** 10.3390/healthcare14213829

**Published:** 2026-11-09

**Authors:** Mercy P. Ontaneda, Marina R. Ramírez

**Affiliations:** Department of Psychology, Universidad Técnica Particular de Loja, Loja 110107, Ecuador; mpontaneda@utpl.edu.ec

**Keywords:** nursing assistants, emotional exhaustion, occupational psychology, sleep quality, psychological inflexibility, work–private-life interference, burnout, Ecuador

## Abstract

Background/Objectives: Evidence concerning emotional exhaustion among nursing assistants in Ecuador remains limited. This exploratory cross-sectional study examined associations of sleep quality, psychological inflexibility, and study-specific work–private-life interference with emotional exhaustion. The focus on emotional exhaustion as the sole outcome was selected during manuscript revision and was not prospectively prespecified. Methods: This analysis included 314 nursing assistants recruited through convenience sampling; 290 had calculable Pittsburgh Sleep Quality Index (PSQI) scores and contributed to PSQI-dependent regression models. Emotional exhaustion was assessed using the nine-item Maslach Burnout Inventory subscale, psychological inflexibility using the AAQ-II, and work–private-life interference using three study-specific binary items. Ordinary least squares regression with HC3 heteroscedasticity-consistent standard errors was evaluated using Python (statsmodels). Results: Of 290 participants with valid PSQI scores, 144 (49.7%) had scores above five. In the main model (R^2^ = 0.432), poorer sleep quality (B = 0.915, 95% CI 0.599–1.231), higher AAQ-II scores (B = 0.196, 95% CI 0.054–0.338), and greater work–private-life interference (B = 3.386, 95% CI 2.454–4.317) were associated with higher emotional exhaustion. Associations persisted after adjustment for age, sex, and shift work. In an exploratory model additionally accounting for concurrent depression, anxiety, and stress symptoms, the AAQ-II association was no longer statistically significant (B = 0.058, *p* = 0.400). Conclusions: Sleep quality and study-specific work–private-life interference were consistently associated with emotional exhaustion. The cross-sectional design, non-probability sampling, and exploratory analyses preclude causal or population-level inferences.

## 1. Introduction

Burnout is an occupational health concern among healthcare workers. Emotional exhaustion, a central component of burnout, refers to the depletion of emotional and psychological resources in response to sustained work demands [1,2]. This study examines emotional exhaustion as a specific continuous outcome rather than estimating the overall prevalence of burnout.

Nursing staff are among the healthcare groups most affected. A meta-analysis of 61 studies from 49 countries estimated a pooled prevalence of burnout symptoms of 11.23% among nurses, with substantial variation across regions, specialties, and measurement approaches [3]. The COVID-19 pandemic added further pressure to nursing work worldwide [4]. Burnout and poor staff well-being have also been associated with poorer patient safety, reduced job performance, poorer quality of care, adverse events, and intention to leave [5,6]. An umbrella review of healthcare workers during the COVID-19 pandemic also documented substantial burdens of sleep disturbance and psychological distress, although its estimates are not specific to Ecuadorian nursing assistants [7].

The Job Demands–Resources model provides a useful framework for understanding burnout. Job demands are primarily related to exhaustion, whereas insufficient job resources are more closely related to disengagement [8,9]. Later developments of the model incorporated personal resources, defined as individual characteristics that may help workers cope with demands [10]. High workload, inadequate staffing, long shifts, and low control have repeatedly been identified as relevant factors in nursing burnout [6]. Work–family conflict, which reduces the time and energy available for recovery, has also been consistently associated with burnout [11]. A meta-analysis additionally links work–family conflict with adverse outcomes across occupational settings [12]. A more recent systematic review similarly documents associations between work–family conflict and mental health, without establishing causality for this sample [13].

Nursing assistants provide direct patient care under demanding occupational conditions, yet their occupational well-being has received less research attention than that of registered nurses. Prior studies have examined nursing assistants’ emotion regulation, burnout, and contributions to care outcomes [14,15]. A systematic review of 28 studies of healthcare assistants identified relatively limited attention to organizational and policy factors [16], and a synthesis of nursing assistants in long-term care emphasized both job characteristics and personal resources [17]. Importantly, sleep, work–family conflict, and mental health have already been examined together in nursing assistants [18]; the present study therefore does not claim novelty for these associations individually. Its contribution is a context-specific, jointly adjusted examination of sleep quality, psychological inflexibility, and a clearly defined study-specific work–private-life interference indicator in relation to emotional exhaustion among Ecuadorian nursing assistants. Considering these factors together may help distinguish occupational and recovery-related correlates from a psychological process whose association could partly reflect concurrent distress. Previous Ecuadorian burnout research provides national context but included a different occupational mix and design [19].

Sleep quality may help explain individual differences in burnout. Adequate sleep is important for cognitive and emotional functioning, whereas disrupted sleep can impair emotion regulation [20]. A meta-analysis in nurses reported a moderate positive correlation between burnout and sleep problems [21]. Longitudinal evidence suggests that insufficient sleep may precede clinical burnout [22], while severe burnout is also associated with disturbed sleep and fatigue [23], indicating that the relationship may be bidirectional. Research on recovery from occupational burnout has also discussed the potential contribution of impaired sleep recovery independently of depressive mood [24].

Healthcare work is frequently organized in shifts, which can disrupt sleep. Shift work disorder is common in occupational samples [25], and high rates have been reported among nurses [26]. Long shifts have also been associated with greater burnout, job dissatisfaction, and intention to leave [27,28]. Given the high prevalence of shift work in the present sample, sleep quality is a plausible correlate of burnout in this group.

Psychological inflexibility refers to difficulty adapting behavior to contextual demands while remaining strongly influenced by internal experiences such as thoughts, emotions, and bodily sensations. It is central to Acceptance and Commitment Therapy and is associated with poorer psychological adaptation and health outcomes [29,30]. Within the Job Demands–Resources framework, psychological flexibility can be conceptualized as a personal resource. Greater psychological flexibility has been associated with lower burnout and compassion fatigue among geriatric nurses [31]. Psychological inflexibility has also been associated with sleep difficulties [32,33,34].

The objective of this study was to examine the cross-sectional associations of sleep quality, psychological inflexibility, and study-specific work–private-life interference with emotional exhaustion among Ecuadorian nursing assistants. The main model included these three predictors; age, sex, and shift work were included in a sensitivity model. The decision to focus this report exclusively on emotional exhaustion was made during manuscript revision and was not prospectively prespecified. Additional adjustment for concurrent DASS-21 depression, anxiety, and stress symptoms was exploratory and post hoc.

## 2. Materials and Methods

### 2.1. Study Design and Participants

This cross-sectional study examined nursing assistants working in public and private healthcare institutions in Ecuador as a subgroup of a broader occupational-health research project. According to the published report from the parent project, 138 healthcare institutions were invited and 79 agreed to participate [35]. These institutional recruitment figures describe the parent project, not the nursing-assistant subgroup. Within participating institutions, eligible nursing assistants who were available during scheduled data collection were invited by evaluators, resulting in a non-probability convenience sample. The subgroup dataset does not identify how many of the 79 institutions contributed nursing assistants, nor the total number of nursing assistants invited; consequently, a subgroup-specific institutional participation count and response rate cannot be calculated.

Participants were eligible if they were actively working as nursing assistants, had at least one year of professional experience, held the corresponding qualifying credential, and provided informed consent. Individuals who did not meet the eligibility criteria, declined participation, or were unavailable during the assessment period were not included in the study. Questionnaires containing sufficient information for study inclusion were retained even when an instrument-specific score could not be calculated. Accordingly, 314 nursing assistants were included in the study, of whom 290 had a computable global PSQI score for PSQI-dependent analyses. No a priori sample-size calculation was performed specifically for this subgroup. Because the total number of nursing assistants invited was not available, a subgroup-specific response rate could not be calculated. The study is reported in accordance with the STROBE Statement [36] (see the STROBE Checklist in Supplementary File S1).

### 2.2. Ethical Considerations

The study was conducted in accordance with the Declaration of Helsinki and was approved by the Human Research Ethics Committee of Universidad Técnica Particular de Loja (CEISH-UTPL), Ecuador, on 14 January 2022 (approval letter CEISH-03-2022; protocol registration code UTPL-CEISH-2022-PI03). Participation was voluntary and uncompensated, confidentiality was guaranteed, and informed consent was obtained before questionnaire completion.

### 2.3. Procedure

Sixty psychologists received 20 h of training from the research team to conduct the assessments [35]. The research team first contacted healthcare institution directors to present the study and request institutional participation; directors of institutions that agreed to participate provided signed letters of acceptance. Survey administrators were organized according to the geographical locations of participating institutions, and assessment dates were scheduled. Potential participants were contacted individually and informed about the research process. Acceptance of individual informed consent was requested as the first step, before any assessment or questionnaire administration; only those who consented proceeded to the assessment. Data were collected between April and August 2022 through a digital questionnaire implemented in ArcGIS Survey123 (version 3.15; Esri, Redlands, CA, USA). Survey123 recorded participant responses but did not automatically calculate PSQI scores; scoring was performed after data export. The study instruments and data-collection procedures were established by the UTPL research team and documented in the project materials.

### 2.4. Instruments

Sociodemographic and work-related characteristics were collected using an ad hoc questionnaire and included age, sex, marital status, work setting, type of institution, type of contract, years of work experience, work schedule, daily working hours, shift work, treatment of patients at risk of death, and perceived availability of institutional resources.

The full Spanish Maslach Burnout Inventory (MBI) was administered in the original 2022 study [1,37]. For the present analysis, emotional exhaustion was operationalized exclusively using its nine-item subscale (item responses 0–6; total score range 0–54), with higher scores indicating more frequent exhaustion symptoms. The subscale showed good internal consistency in this sample (Cronbach’s α = 0.857). Sample-specific internal consistency was also examined for the other administered MBI dimensions: depersonalization (Cronbach’s α = 0.556; one-factor omega estimate = 0.583) and personal accomplishment (α = 0.870; omega = 0.877). For emotional exhaustion, the corresponding one-factor omega estimate was 0.870. These omega values were estimated using a one-factor model for each subscale and should not be interpreted as evidence of factorial validity. The low reliability of depersonalization provided an additional measurement-related reason for caution when considering that dimension; personal accomplishment showed adequate reliability. The focus on emotional exhaustion was informed by its conceptual relevance to the study questions and its adequate internal consistency, and was selected during manuscript revision rather than prespecified.

Psychological inflexibility was measured with the Ecuadorian version [38] of the Acceptance and Action Questionnaire-II (AAQ-II) [39]. The seven items are rated from 1 (never) to 7 (always), with total scores ranging from 7 to 49; higher scores indicate greater psychological inflexibility. In the present sample, Cronbach’s α was 0.923.

Sleep quality was assessed using the Pittsburgh Sleep Quality Index (PSQI) [40] in its Spanish adaptation [41]. Seven component scores, each ranging from 0 to 3, are summed to produce a global score ranging from 0 to 21; higher scores indicate poorer sleep quality, and scores above 5 indicate poor sleep quality [40]. In the analytical dataset, the seven-component total (PSQI_7C_TOTAL) was calculable for 290 participants and was used in the PSQI-dependent analyses. Cronbach’s α across the seven recorded components was 0.697. An arithmetic check confirmed that the total equaled the sum of the seven recorded component scores for these 290 participants, each component being within the expected 0–3 range. The historical field labeled PSQI excluded component 4 (habitual sleep efficiency), accounting for differences between the two totals in 121 of the 290 calculable records. The historical field was retained for traceability; this check established consistency of the recorded component sum but did not independently revalidate every component against the original questionnaire responses.

Work–private-life interference was operationalized using three dichotomous (yes/no) questions from the ad hoc sociodemographic and occupational questionnaire administered in the parent research project. The questions addressed (i) whether work consumed so much energy that it negatively affected private life (dataset variable SD2), (ii) whether work took so much time that it negatively affected private life (SD3), and (iii) whether respondents experienced situations requiring them to be at work and at home simultaneously (SD4). The analytic indicator was calculated as SD2 + SD3 + SD4, with each item coded 0 = No and 1 = Yes (possible composite range: 0–3); higher scores indicate greater work–private-life interference. All 314 records had complete values for these items. Its internal consistency was Cronbach’s α = 0.705 (algebraically equivalent to KR-20 for binary items). This study-specific indicator was not an independently validated psychometric scale and should not be interpreted as a comprehensive multidimensional work–family conflict measure. The project questionnaire reproduced in the parent project teaching guide documents the original Spanish item wording and dichotomous response options (Sí/No): SD2, “¿Siente que su trabajo consume tanta energía que tiene un efecto negativo en su vida privada?” (English translation: “Do you feel that your work consumes so much energy that it has a negative effect on your private life?”); SD3, “¿Siente que su trabajo toma tanto tiempo que tiene un efecto negativo en su vida privada?” (English translation: “Do you feel that your work takes up so much time that it has a negative effect on your private life?”); and SD4, “¿Hay momentos en los que necesita estar en el trabajo y en casa al mismo tiempo?” (English translation: “Are there times when you need to be at work and at home at the same time?”). These item wordings and response options are documented in the study project guide. Item-level endorsement frequencies in the full sample (*N* = 314) were 98/314 (31.2%) for SD2, 74/314 (23.6%) for SD3, and 189/314 (60.2%) for SD4; among the 290 complete cases in the primary model, corresponding frequencies were 89/290 (30.7%), 67/290 (23.1%), and 177/290 (61.0%), respectively. These frequencies reflect the recorded responses. Detailed item-level frequencies are provided in Supplementary Table S1.

Depression, Anxiety and Stress Scales (DASS-21). Symptoms of depression, anxiety and stress were assessed using 21 items corresponding to the Ecuadorian adaptation of the Depression, Anxiety and Stress Scales (DASS-21), described for the related healthcare-worker project [35]. Each of its three dimensions comprises seven items rated from 0 to 3. In the nursing-assistant data, each dimension was calculated as the sum of its seven items (raw score range: 0–21; scores were not doubled). Cronbach’s α was 0.884 for depression, 0.884 for anxiety and 0.877 for stress in the nursing-assistant sample. These dimensions were used exclusively in exploratory post hoc sensitivity analyses rather than the original main regression specification.

### 2.5. Statistical Analysis

An exploratory complete-case comparison assessed differences between nursing assistants with computable seven-component PSQI global scores (*n* = 290) and those without computable scores (*n* = 24). In the final analytical dataset, non-calculable seven-component PSQI global scores were blank. Continuous characteristics were summarized using means and sample standard deviations and compared using two-sided Welch independent-samples t-tests. A supplementary Mann–Whitney U test was reported for age. Available categorical characteristics were summarized using counts and percentages and compared using two-sided Fisher exact tests, given the small incomplete-PSQI subgroup. All comparisons were exploratory, without multiplicity adjustment, and were not used to select regression covariates or change the original models. Reasons for non-computable PSQI scores were summarized from the post-export analytical scoring-status variable (Supplementary Table S2). No missing PSQI component values were imputed.

Statistical analyses were conducted using IBM SPSS Statistics, version 26.0 (IBM Corp., Armonk, NY, USA), and Python, version 3.13.5 (Python Software Foundation, Beaverton, OR, USA), with the statsmodels library (version 0.14.6, NumFOCUS, Inc, Austin, TX, USA). SPSS was used for descriptive summaries, internal-consistency estimates (Cronbach’s α), and bivariate analyses using Spearman correlations. The regression models were independently reproduced in Python using ordinary least squares with HC3 heteroscedasticity-consistent robust standard errors; this reproduction confirmed the reported HC3 regression estimates. Continuous variables were summarized using means and standard deviations, together with medians and interquartile ranges, and categorical variables using frequencies and percentages. Because burnout scores were not normally distributed, bivariate associations were examined using Spearman correlations.

For the primary regression model, two-sided *p*-values and 95% confidence intervals based on HC3 heteroscedasticity-consistent standard errors used the asymptotic standard normal reference distribution. Standardized coefficients (β) were computed as the unstandardized coefficient multiplied by the sample standard deviation of the predictor and divided by the sample standard deviation of emotional exhaustion. The primary regression used 290 complete cases. The original questionnaire asked only whether participants worked in a public or private healthcare institution; it did not request the name or identity of the institution. Consequently, within-institution clustering could not be assessed, and the HC3 confidence intervals and *p*-values rely on an independence assumption that could not be verified at the institution level.

The decision to focus the present report on emotional exhaustion was based on its theoretical relevance to occupational demands, sleep quality, psychological inflexibility, and work–private-life interference, and was supported by its adequate internal consistency in this sample (Cronbach’s α = 0.857; one-factor omega estimate = 0.870). Depersonalization showed lower internal consistency (α = 0.556; omega estimate = 0.583), whereas personal accomplishment also demonstrated adequate internal consistency (α = 0.870; omega estimate = 0.877). Therefore, low reliability was not a reason to exclude both other MBI dimensions. The exhaustion-only focus was adopted during manuscript revision and was not prospectively prespecified. These sample-specific reliability estimates do not establish factorial validity, and the focused analysis should not be interpreted as an assessment of burnout as a whole.

Age, sex, and shift work were included in a sensitivity model as demographic and work-schedule characteristics potentially relevant to sleep quality and emotional exhaustion. This parsimonious adjustment was intended to assess the stability of the associations observed in the primary model, rather than to provide comprehensive control for confounding. Adjustment for concurrent DASS-21 symptoms was conducted separately as an exploratory post hoc analysis. 

Exploratory post hoc models additionally included age, sex, shift work, and the DASS-21 depression, anxiety and stress subscales along with PSQI, AAQ-II and work–private-life interference (*n* = 290). The supplementary models address sensitivity of the AAQ-II associations to contemporaneous distress rather than a prespecified causal confounder set. DASS-21 subscale variance inflation factors were approximately 4.19–5.17; the subscales may overlap with one another and with the AAQ-II. An additional sensitivity comparison substituted the summed DASS-21 symptom score for the three subscales. Non-calculable seven-component PSQI global scores were excluded from PSQI-dependent analyses. In the survey export, GENERO was coded 1 = male and 2 = female; for covariate-adjusted models it was expressed as a female indicator (1 = female, 0 = male), an intercept-only recoding that leaves the sex slope unchanged. Because these symptom dimensions were measured concurrently with the outcome, their inclusion should not be interpreted as establishing confounding control or a causal pathway.

As the full MBI was administered in the parent study but this focused report uses only emotional exhaustion, internal consistency for the nine-item emotional exhaustion subscale (Cronbach’s α = 0.857) is reported as the directly relevant reliability estimate. Reliability-generalization research on the MBI has documented that reliability estimates vary across studies and analytic approaches [42,43]. Accordingly, the sample-specific Cronbach’s α is reported here rather than assuming that estimates from other populations apply to Ecuadorian nursing assistants. The study does not establish the full-scale factorial validity of the emotional exhaustion subscale in this subgroup.

## 3. Results

### 3.1. Participant Characteristics

A total of 314 nursing assistants were included (Table 1). Their mean age was 35.40 years (SD = 9.38; range, 18–63), and median work experience was 6 years (IQR, 3–10). Most participants were female (82.2%), worked in urban areas (84.4%) and public institutions (56.7%), held a permanent appointment (52.5%), worked full-time (78.0%), and reported shift work (78.7%).

### 3.2. Descriptive Statistics

Of the 314 participants, 290 (92.4%) had a computable PSQI global score and 24 (7.6%) did not. The analytical scoring-status variable classified the 24 non-computable scores as time in bed equal to zero (*n* = 12), invalid sleep duration (*n* = 11), or invalid bedtime/wake-time (*n* = 1); these categories describe the scoring-status indicators recorded in the analytical dataset. In exploratory comparisons, mean age was 38.67 ± 8.48 years among participants without a computable PSQI score and 35.13 ± 9.41 years among those with a computable score (Welch *p* = 0.062; supplementary Mann–Whitney U = 2567.5, *p* = 0.033). Mean emotional exhaustion scores were 10.50 ± 8.53 and 12.83 ± 10.02, respectively (Welch *p* = 0.214). Fisher exact comparisons of female sex (*p* = 0.402) and shift work (*p* = 1.000) did not indicate statistically significant differences. The full descriptive comparisons, including other available continuous and categorical characteristics, are presented in Supplementary Table S2. These post hoc comparisons were exploratory; the small incomplete-PSQI subgroup limits precision, and the analyses cannot establish that missing PSQI scores were missing at random.

The mean emotional exhaustion score was 12.66 (SD = 9.92). Among the 290 participants with computable PSQI global scores, 144 (49.7%) were classified as poor sleepers. Descriptive statistics and internal consistency estimates are shown in Table 2.

### 3.3. Correlations

Poorer sleep quality, greater psychological inflexibility, and higher scores on the work–private-life interference indicator were each associated with higher emotional exhaustion. The work–private-life interference indicator showed the largest bivariate correlation with emotional exhaustion (ρ = 0.514). Table 3 presents correlations among the four variables included in this focused report.

### 3.4. Main Outcome: Emotional Exhaustion

The emotional exhaustion model explained 43.2% of outcome variance (R^2^ = 0.432). Poorer sleep quality (B = 0.915, 95% CI 0.599–1.231, *p* < 0.001), greater psychological inflexibility (B = 0.196, 95% CI 0.054–0.338, *p* = 0.007), and higher scores on the study-specific work–private-life interference indicator (B = 3.386, 95% CI 2.454–4.317, *p* < 0.001) were each associated with higher exhaustion in the original mutually adjusted model (Table 4).

Exploratory model diagnostics and functional-form sensitivity analyses were performed on the same 290 complete cases. The Breusch–Pagan test suggested heteroscedasticity (*p* = 0.039), and 27 observations exceeded the screening threshold of 4/n for Cook’s distance. A categorical specification of the 0–3 work–private-life interference indicator produced a similar model fit (R^2^ = 0.4341 versus 0.4318 for the linear specification; robust departure-from-linearity test *p* = 0.700). Exploratory quadratic terms suggested possible nonlinearity for PSQI (*p* = 0.0018), particularly requiring caution at sparsely represented high scores. Excluding SD2 from the interference indicator in a separate exploratory sensitivity model retained a positive association (B = 4.643, 95% CI 3.341–5.944, R^2^ = 0.4211; HC3, t-based interval). These post hoc findings do not replace the primary model estimates and do not establish the validity of the reduced indicator; further details are provided in Supplementary File S2.

### 3.5. Covariate-Adjusted Sensitivity Analyses

In the model additionally adjusting for age, sex, and shift work (*n* = 290), PSQI global score (B = 0.941, 95% CI 0.624–1.257, *p* < 0.001), AAQ-II score (B = 0.186, 95% CI 0.054–0.317, *p* = 0.006), and the study-specific work–private-life interference indicator (B = 3.298, 95% CI 2.371–4.226, *p* < 0.001) remained associated with emotional exhaustion. Model R^2^ was 0.4318 in the original model and 0.4474 after adding these covariates, an absolute increase of 0.0156 (1.56 percentage points of explained variance). The direction and approximate magnitude of the focal coefficients were broadly stable. This analysis assesses sensitivity to the measured covariates; it does not establish predictive performance in new samples or eliminate unmeasured confounding (Supplementary Table S3).

### 3.6. Exploratory Post Hoc Model Accounting for Psychological Distress

In an exploratory post hoc model including PSQI global score, AAQ-II, the study-specific work–private-life interference indicator, age, sex, shift work, and the three DASS-21 symptom subscales (*n* = 290), the AAQ-II coefficient was attenuated and its confidence interval included zero (B = 0.0577, 95% CI −0.077 to 0.192, *p* = 0.400). The full model had R^2^ = 0.5039. In the nested comparison, R^2^ increased from 0.5025 without AAQ-II to 0.5039 with AAQ-II (ΔR^2^ = 0.0014; 0.14 percentage points), indicating a small incremental in-sample contribution conditional on the other predictors (Supplementary Table S4). Replacing the three DASS-21 subscales with an aggregated symptom score also yielded a non-significant AAQ-II coefficient (B = 0.0481, 95% CI −0.0830 to 0.1791, *p* = 0.4723; R^2^ = 0.4965; ΔR^2^ attributable to AAQ-II = 0.0010). These post hoc findings do not establish mediation, construct redundancy, or the absence of an underlying association; contemporaneous psychological measures may share variance and the cross-sectional design cannot establish temporal ordering (Table 5; Supplementary Table S4).

## 4. Discussion

In this sample of Ecuadorian nursing assistants, poorer sleep quality, higher psychological inflexibility, and greater work–private-life interference were each associated with higher emotional exhaustion in the primary mutually adjusted model (R^2^ = 0.432). The associations of sleep quality and work–private-life interference also persisted in the demographic-adjusted sensitivity analysis. In contrast, the association of psychological inflexibility was attenuated and no longer statistically significant after exploratory adjustment for concurrent depression, anxiety, and stress symptoms. These findings provide context-specific evidence from a direct-care occupational group and suggest that the interpretation of psychological inflexibility depends on which dimensions of psychological distress are considered. Because the outcome focus and DASS-21 analyses were selected during manuscript revision, these findings should be treated as exploratory rather than confirmatory.

The findings can be considered within the Job Demands–Resources framework, in which sustained demands and limited recovery opportunities may accompany emotional exhaustion [8,9,10]. Work–private-life interference may reflect competing occupational and nonoccupational demands, and poorer sleep quality may indicate disrupted recovery. Psychological inflexibility concerns responses to internal experiences and is not equivalent to an organizational resource directly measured in this study. The concurrent measurements and alternative adjustment sets do not establish directionality or underlying mechanisms.

Poor sleep quality was common among participants and remained associated with greater emotional exhaustion across the primary and demographic-adjusted models. This finding is consistent with previous research linking sleep disturbances and burnout-related outcomes in nursing personnel [20,21,22,23]. However, the present study extends this discussion to Ecuadorian nursing assistants while examining sleep quality alongside psychological inflexibility and work–private-life interference. Because sleep and exhaustion were measured concurrently, the findings cannot determine whether disturbed sleep precedes emotional exhaustion, follows occupational strain, or reflects a reciprocal relationship.

The attenuation of the AAQ-II association after accounting for concurrent depression, anxiety, and stress symptoms qualifies its apparent contribution in the primary model. Rather than demonstrating that psychological inflexibility is unrelated to emotional exhaustion, this pattern suggests that its association may partly reflect variance shared with concurrent psychological distress. Previous nursing research has linked psychological flexibility with burnout and mental health [44], whereas concerns about AAQ-II discriminant validity [45] and conceptual overlap between burnout and depressive symptoms [46] provide reasons for caution. These findings highlight the importance of distinguishing psychological inflexibility from concurrent distress in future longitudinal studies. The present cross-sectional analyses cannot establish mediation or causal pathways.

Work–private-life interference showed a consistent positive association with emotional exhaustion. Within the Job Demands–Resources perspective, competing occupational and personal demands may be relevant to understanding limited opportunities for recovery [8,9,10,11]. However, the study-specific indicator differs from independently validated multidimensional work–family conflict measures. In particular, the item referring to work-related energy depletion (SD2) may overlap conceptually with emotional exhaustion and contribute to the observed association. Therefore, the findings should be interpreted as exploratory evidence concerning a study-specific indicator rather than confirmation of an independent causal mechanism.

### 4.1. Practical Implications

The findings suggest that sleep quality and work–private-life interference are relevant domains for occupational-health assessment among Ecuadorian nursing assistants. Nursing managers and healthcare organizations could consider examining shift schedules, opportunities for rest and recovery, and competing work–family responsibilities when assessing staff well-being. Such assessments may help identify working conditions that warrant closer attention, particularly where employees report poor sleep or difficulties reconciling occupational and personal demands. These implications should not be interpreted as evidence that specific scheduling changes or organizational interventions would reduce emotional exhaustion. The cross-sectional design does not establish temporal or causal relationships, and no intervention was evaluated. Furthermore, the attenuation of the AAQ-II association after adjustment for concurrent psychological distress indicates that recommendations concerning psychological flexibility should be based on additional evidence rather than the present findings alone. Prospective and intervention studies are needed to determine whether modifying occupational conditions improves emotional exhaustion among nursing assistants.

### 4.2. Limitations

Several limitations qualify these findings. First, the cross-sectional design prevents temporal or causal inference. Second, all main measures were self-reported, raising the possibility of shared-method and response biases. Third, convenience sampling, unavailable subgroup recruitment denominators, and potential clustering within institutions limit representativeness and the precision of conventional individual-level inferences; HC3 robust errors address heteroscedasticity but not institution-level dependence. Fourth, the report focuses only on the MBI emotional exhaustion subscale; its internal consistency (α = 0.857) does not establish factorial validity. Fifth, 24 of 314 participants lacked calculable PSQI scores and were excluded from PSQI-dependent regressions. Exploratory comparisons of participants with computable and non-computable PSQI global scores (Supplementary Table S2) cannot demonstrate that the missing data were random. Sixth, the three binary items measuring work–private-life interference constitute a study-specific indicator without independent construct validation. Seventh, residual confounding is possible despite demographic and shift-work adjustment. Eighth, the exhaustion-only reporting focus and DASS-21 adjustments were post hoc, and multiple exploratory analyses were not designed as confirmatory tests. Finally, AAQ-II and DASS-21 scores may capture overlapping aspects of psychological responding, while correlations among the DASS-21 subscales complicate interpretation of the attenuated AAQ-II coefficient. These limitations argue against treating the exploratory result as proof of construct redundancy or absence of an underlying association.

## 5. Conclusions

In this non-probability sample of Ecuadorian nursing assistants, poorer sleep quality and greater study-specific work–private-life interference were associated with higher emotional exhaustion. Psychological inflexibility was also associated with emotional exhaustion in the primary model, but this association was attenuated after exploratory adjustment for concurrent psychological distress. These findings highlight the importance of considering sleep, occupational demands, and overlapping psychological constructs when investigating emotional exhaustion. The cross-sectional design, study-specific interference measure, and limitations related to institutional clustering and residual confounding preclude causal interpretation. Prospective, multisite studies using independently validated measures are needed to clarify these relationships and inform occupational-health research.

## Figures and Tables

**Table 1:** Sociodemographic and work characteristics of nursing assistants (*N* = 314).

Characteristic	Value
Age, years, mean ± SD	35.40 ± 9.38
Work experience, years, median (IQR)	6 (3–10)
Sex, *n* (%)	
Female	258 (82.2)
Male	56 (17.8)
Marital status, *n* (%)	
Single	146 (46.5)
Married/free union	130 (41.4)
Divorced	33 (10.5)
Widowed	5 (1.6)
Work schedule, *n* (%)	
Full-time	245 (78.0)
Part-time	69 (22.0)
Shift work, *n* (%)	
Yes	247 (78.7)
No	67 (21.3)
Area of work, *n* (%)	
Urban	265 (84.4)
Rural	49 (15.6)
Type of institution, *n* (%)	
Public	178 (56.7)
Private	136 (43.3)
Type of contract, *n* (%)	
Permanent appointment	165 (52.5)
Provisional appointment	54 (17.2)
Occasional services	53 (16.9)
Discretionary appointment	23 (7.3)
Other	19 (6.1)
Treated patients at risk of death in the previous year, *n* (%)	
Yes	252 (80.3)
No	62 (19.7)
Institution provides the resources needed, *n* (%)	
Fully	1 (0.3)
Moderately	33 (10.5)
A little	136 (43.3)
Not at all	144 (45.9)

IQR, interquartile range; SD, standard deviation.

**Table 2:** Descriptive statistics and internal consistency of the study variables.

Variable	*N*	Mean ± SD	Median (IQR)	Observed Range	Cronbach’s α
Emotional exhaustion	314	12.66 ± 9.92	10 (5–18)	0–46	0.857
AAQ-II	314	14.17 ± 8.01	12 (8–17)	7–46	0.923
PSQI global score	290	6.06 ± 3.64	5 (3–8)	0–20	0.697
Work–private-life interference (SD2 + SD3 + SD4)	314	1.15 ± 1.10	1 (0–2)	0–3	0.705

Note. IQR indicates the first and third quartiles (Q1–Q3) shown in parentheses; ranges are observed minimum–maximum values. AAQ-II, Acceptance and Action Questionnaire-II; IQR, interquartile range; PSQI, Pittsburgh Sleep Quality Index; SD, standard deviation.

**Table 3:** Spearman correlations among study variables.

Variable	1	2	3	4
1. Emotional exhaustion	—			
2. AAQ-II	0.438 ***	—		
3. PSQI global score	0.451 ***	0.423 ***	—	
4. Work–private-life interference	0.514 ***	0.322 ***	0.339 ***	—

Correlations involving PSQI are based on *n* = 290; all other correlations are based on *n* = 314. *** *p* < 0.001.

**Table 4:** HC3-robust multiple linear regression for emotional exhaustion (*n* = 290).

Outcome/Predictor	B	95% CI	β	*p*	R^2^
Emotional exhaustion					0.432
PSQI global score	0.915	0.599 to 1.231	0.332	<0.001	
AAQ-II	0.196	0.054 to 0.338	0.157	0.007	
Work–private-life interference indicator	3.386	2.454 to 4.317	0.371	<0.001	

B, unstandardized coefficient; β, standardized coefficient. Confidence intervals and *p*-values are based on HC3 robust standard errors. AAQ-II, Acceptance and Action Questionnaire-II; PSQI, Pittsburgh Sleep Quality Index. Primary-model 95% confidence intervals and *p*-values use the asymptotic standard normal reference distribution.

**Table 5:** Exploratory post hoc HC3-robust regression of emotional exhaustion, adjusted for PSQI, the study-specific work–private-life interference indicator, age, sex, shift work and DASS-21 symptom subscales.

Outcome	AAQ-II B (95% CI)	AAQ-II *p*	Model R^2^	*n*
Emotional exhaustion	0.0577 (−0.077 to 0.192)	0.400	0.5039	290

## Data Availability

The anonymized data supporting the findings of this study are not publicly available to protect participant confidentiality. Data may be requested from the corresponding author, Marina R. Ramírez (mrramirez@utpl.edu.ec), upon reasonable request and are subject to prior authorization and applicable ethical and institutional requirements.

## Supplementary Materials

The following supporting information can be downloaded at: https://www.mdpi.com/article/10.3390/healthcare14213829/s1, Table S1: Endorsement frequencies of the three study-specific work–private-life interference items among Ecuadorian nursing assistants (*N* = 314); Table S2: Exploratory comparison by PSQI score computability; Table S3: Covariate-adjusted sensitivity model for emotional exhaustion; Table S4: Exploratory post hoc sensitivity models including concurrent psychological distress (*n* = 290); File S1: STROBE Cross-Sectional Reporting Checklist—Reporting Cross-Reference; File S2: Exploratory regression diagnostics and sensitivity analyses (Table S5: Model diagnostics and functional form; File S2.1: Cook’s distance sensitivity check; File S2.2: PSQI Scoring and Data-Cleaning Appendix).

## References

[1] Maslach C., Jackson S.E., Leiter M.P. (1996). Maslach Burnout Inventory Manual.

[2] Maslach C., Leiter M.P. (2016). Understanding the burnout experience: Recent research and its implications for psychiatry. World Psychiatry.

[3] Woo T., Ho R., Tang A., Tam W. (2020). Global prevalence of burnout symptoms among nurses: A systematic review and meta-analysis. J. Psychiatr. Res..

[4] Fekih-Romdhane F., Harb F., Al Banna S., Obeid S., Hallit S. (2025). Prevalence and risk factors of burnout symptoms among nurses during the COVID-19 pandemic: An updated systematic review and meta-analysis. Hum. Resour. Health.

[5] Hall L.H., Johnson J., Watt I., Tsipa A., O’Connor D.B. (2016). Healthcare staff wellbeing, burnout, and patient safety: A systematic review. PLoS ONE.

[6] Dall’Ora C., Ball J., Reinius M., Griffiths P. (2020). Burnout in nursing: A theoretical review. Hum. Resour. Health.

[7] Al Maqbali M., Alsayed A., Hughes C., Hacker E., Dickens G.L. (2024). Stress, anxiety, depression and sleep disturbance among healthcare professional during the COVID-19 pandemic: An umbrella review of 72 meta-analyses. PLoS ONE.

[8] Demerouti E., Nachreiner F., Bakker A.B., Schaufeli W.B. (2001). The job demands-resources model of burnout. J. Appl. Psychol..

[9] Bakker A.B., Demerouti E. (2007). The Job Demands-Resources model: State of the art. J. Manag. Psychol..

[10] Bakker A.B., Demerouti E. (2017). Job demands–resources theory: Taking stock and looking forward. J. Occup. Health Psychol..

[11] Allen T.D., Herst D.E., Bruck C.S., Sutton M. (2000). Consequences associated with work-to-family conflict: A review and agenda for future research. J. Occup. Health Psychol..

[12] Amstad F.T., Meier L.L., Fasel U., Elfering A., Semmer N.K. (2011). A meta-analysis of work–family conflict and various outcomes with a special emphasis on cross-domain versus matching-domain relations. J. Occup. Health Psychol..

[13] Liu D., Ni Y., Ma T.S.W., Fan X., Zhan Y., Lam I.H.L., Lui M.C.L., Chandola T., Kawachi I., Cuijpers P. (2026). Work-family conflict and mental health: A systematic review and meta-analysis. PLoS Med..

[14] Bamonti P., Conti E., Cavanagh C., Gerolimatos L., Gregg J., Goulet C., Pifer M., Edelstein B. (2019). Coping, cognitive emotion regulation, and burnout in long-term care nursing staff: A preliminary study. J. Appl. Gerontol..

[15] Li X., Dorstyn D., Mpofu E., O’Neill L., Li Q., Zhang C., Ingman S. (2021). Nursing assistants and resident satisfaction in long-term care: A systematic review. Geriatr. Nurs..

[16] Norful A.A., Brewer K.C., Cahir K.M., Dierkes A.M. (2024). Individual and organizational factors influencing well-being and burnout amongst healthcare assistants: A systematic review. Int. J. Nurs. Stud. Adv..

[17] Huang S., Yau S.Y., Lee Y.K.L., Song J., Guo Y., Dong D. (2025). Job characteristics, personal characteristics and well-being of nursing assistants in long-term care facilities: A mixed methods systematic review and narrative synthesis. Int. J. Nurs. Stud..

[18] Zhang Y., Punnett L., Nannini A. (2017). Work-Family Conflict, Sleep, and Mental Health of Nursing Assistants Working in Nursing Homes. Workplace Health Saf..

[19] Ramírez M.R., Otero P., Blanco V., Ontaneda M.P., Díaz O., Vázquez F.L. (2018). Prevalence and correlates of burnout in health professionals in Ecuador. Compr. Psychiatry.

[20] Palmer C.A., Alfano C.A. (2017). Sleep and emotion regulation: An organizing, integrative review. Sleep Med. Rev..

[21] Membrive-Jiménez M.J., Gómez-Urquiza J.L., Suleiman-Martos N., Velando-Soriano A., Ariza T., De la Fuente-Solana E.I., Cañadas-De la Fuente G.A. (2022). Relation between burnout and sleep problems in nurses: A systematic review with meta-analysis. Healthcare.

[22] Söderström M., Jeding K., Ekstedt M., Perski A., Åkerstedt T. (2012). Insufficient sleep predicts clinical burnout. J. Occup. Health Psychol..

[23] Ekstedt M., Söderström M., Åkerstedt T., Nilsson J., Søndergaard H.P., Perski A. (2006). Disturbed sleep and fatigue in occupational burnout. Scand. J. Work Environ. Health.

[24] Sonnenschein M., Sorbi M.J., van Doornen L.J., Schaufeli W.B., Maas C.J. (2007). Evidence that impaired sleep recovery may complicate burnout improvement independently of depressive mood. J. Psychosom. Res..

[25] Pallesen S., Bjorvatn B., Waage S., Harris A., Sagoe D. (2021). Prevalence of shift work disorder: A systematic review and meta-analysis. Front. Psychol..

[26] Haile K.K., Asnakew S., Waja T., Kerbih H.B. (2019). Shift work sleep disorders and associated factors among nurses at federal government hospitals in Ethiopia: A cross-sectional study. BMJ Open.

[27] Dall’Ora C., Griffiths P., Ball J., Simon M., Aiken L.H. (2015). Association of 12 h shifts and nurses’ job satisfaction, burnout and intention to leave: Findings from a cross-sectional study of 12 European countries. BMJ Open.

[28] Stimpfel A.W., Sloane D.M., Aiken L.H. (2012). The longer the shifts for hospital nurses, the higher the levels of burnout and patient dissatisfaction. Health Aff..

[29] Hayes S.C., Luoma J.B., Bond F.W., Masuda A., Lillis J. (2006). Acceptance and Commitment Therapy: Model, processes and outcomes. Behav. Res. Ther..

[30] Kashdan T.B., Rottenberg J. (2010). Psychological flexibility as a fundamental aspect of health. Clin. Psychol. Rev..

[31] Sarabia-Cobo C., Pérez V., de Lorena P., Fernández-Rodríguez Á., González-López J.R., González-Vaca J. (2021). Burnout, compassion fatigue and psychological flexibility among geriatric nurses: A multicenter study in Spain. Int. J. Environ. Res. Public Health.

[32] Kato T. (2016). Impact of psychological inflexibility on depressive symptoms and sleep difficulty in a Japanese sample. SpringerPlus.

[33] Kato T. (2020). Effect of psychological inflexibility on depressive symptoms and sleep disturbance among Japanese young women with chronic pain. Int. J. Environ. Res. Public Health.

[34] Zalamea-Delgado M.P., Jiménez-López E., Mesas A.E., Yáñez-Sepúlveda R., Gutiérrez-Espinoza H., Olivares-Arancibia J., Ezzatvar Y., Stubbs B., Smith L., Ridgell L. (2026). Exploring the association between psychological inflexibility, experiential avoidance and sleep-related problems among adolescents: The EHDLA study. Healthcare.

[35] Ramírez M.R., Ontaneda M.P., Otero P., Ortega-Jiménez D., Blanco V., Vázquez F.L. (2025). Burnout, associated factors, and mental health measures among Ecuadorian physicians: A cross-sectional study. J. Clin. Med..

[36] von Elm E., Altman D.G., Egger M., Pocock S.J., Gøtzsche P.C., Vandenbroucke J.P., STROBE Initiative (2007). The Strengthening the Reporting of Observational Studies in Epidemiology (STROBE) Statement: Guidelines for Reporting Observational Studies. PLoS Med..

[37] Maslach C., Jackson S.E. (1997). MBI: Inventario Burnout de Maslach: Síndrome del Quemado por Estrés Laboral Asistencial: Manual.

[38] Paladines-Costa B., López-Guerra V., Ruisoto P., Vaca-Gallegos S., Cacho R. (2021). Psychometric properties and factor structure of the Spanish version of the Acceptance and Action Questionnaire-II (AAQ-II) in Ecuador. Int. J. Environ. Res. Public Health.

[39] Bond F.W., Hayes S.C., Baer R.A., Carpenter K.M., Guenole N., Orcutt H.K., Waltz T., Zettle R.D. (2011). Preliminary psychometric properties of the Acceptance and Action Questionnaire–II: A revised measure of psychological inflexibility and experiential avoidance. Behav. Ther..

[40] Buysse D.J., Reynolds C.F., Monk T.H., Berman S.R., Kupfer D.J. (1989). The Pittsburgh Sleep Quality Index: A new instrument for psychiatric practice and research. Psychiatry Res..

[41] Macías Fernández J.A., Royuela Rico A. (1996). La versión española del Índice de Calidad de Sueño de Pittsburgh. Inf. Psiquiátr..

[42] Aguayo R., Vargas C., de la Fuente E.I., Lozano L.M. (2011). A meta-analytic reliability generalization study of the Maslach Burnout Inventory. Int. J. Clin. Health Psychol..

[43] Aguayo-Estremera R., Cañadas-De la Fuente G.R., Ariza-Castilla T., Ortega-Campos E., Gómez-Urquiza J.L., Romero-Béjar J.L., De la Fuente-Solana E.I. (2024). A comparison of univariate and meta-analytic structural equation modeling approaches to reliability generalization applied to the Maslach Burnout Inventory. Front. Psychol..

[44] Chong Y.Y., Frey E., Chien W.T., Cheng H.Y., Gloster A.T. (2023). The role of psychological flexibility in the relationships between burnout, job satisfaction, and mental health among nurses in combatting COVID-19: A two-region survey. J. Nurs. Scholarsh..

[45] Wolgast M. (2014). What does the Acceptance and Action Questionnaire (AAQ-II) really measure?. Behav. Ther..

[46] Bianchi R., Schonfeld I.S., Laurent E. (2015). Burnout–depression overlap: A review. Clin. Psychol. Rev..

