# Supplementary material for: Sleep Quality, Psychological Inflexibility, and Work–Private-Life Interference in Relation to Emotional Exhaustion Among Ecuadorian Nursing Assistants: A Cross-Sectional Study
Source: Healthcare (Basel). 2026 Nov 9;14(21):3829. doi: 10.3390/healthcare14213829 (PMC13763159; doi:10.3390/healthcare14213829)
Supplement: Supplementary file 1 [file healthcare-14-03829-s001.zip › healthcare-4666063-supplementary.pdf]

Supplementary Materials

Table S1. Endorsement frequencies of the three study-specific work–private-life interference items among Ecuadorian nursing assistants (N = 314).

Item	Item content (English summary)	Yes, n (%)	No, n (%)	Valid n
SD2	Work consumed so much energy that it negatively affected private life	98 (31.2)	216 (68.8)	314
SD3	Work took so much time that it negatively affected private life	74 (23.6)	240 (76.4)	314
SD4	Situations requiring presence at work and at home simultaneously	189 (60.2)	125 (39.8)	314

Note. Values were calculated directly from the final N = 314 analytical dataset. Items were coded 0 = No and 1 = Yes. The English descriptions summarize the item content and are not presented as verbatim translations of the original Spanish questionnaire. Percentages use all 314 participants as the denominator; no item-level missing values were found for SD2–SD4.

Table S2. Exploratory comparison by PSQI score computability

Nursing assistants with a calculable seven-component PSQI global score (n = 290) and those without a calculable score (n = 24), from the full analytical sample (N = 314).

Characteristic	Calculable PSQI (n = 290)	Non-calculable PSQI (n = 24)	p-value
Age, years, mean ± SD	35.13 ± 9.41	38.67 ± 8.48	0.062*
Sex: female	240 (82.8%)	18 (75.0%)	0.402
Shift work: yes	228 (78.6%)	19 (79.2%)	1.000
Institution type: public	163 (56.2%)	15 (62.5%)	0.670
Work area: urban	242 (83.4%)	23 (95.8%)	0.145

Age, Mann–Whitney U sensitivity test: p = 0.033.

Notes. Values are n (%) unless otherwise indicated. *Welch unequal-variance two-sample t-test. Two-sided Fisher exact tests were used for the four binary characteristics. The Mann–Whitney U test for age was an additional exploratory comparison. Tests were not adjusted for multiplicity; these comparisons cannot establish that PSQI non-computability was random. Coding of sex, shift work, institution type, and work area follows the analytical dataset and the manuscript Table 1. The survey recorded public/private institution type, not individual institution identity.

Source: Study analytical dataset (N = 314); seven-component PSQI score-computability classification.

Table S3. Covariate-adjusted sensitivity model for emotional exhaustion

Ordinary least squares regression with HC3 heteroscedasticity-consistent standard errors (n = 290).

Predictor	B	95% CI	p-value
Intercept	6.213	1.803 to 10.622	0.006
PSQI global score	0.941	0.624 to 1.257	<0.001
AAQ-II total score	0.186	0.054 to 0.317	0.006
Work–private-life interference (0–3)	3.298	2.371 to 4.226	<0.001
Age (years)	-0.104	-0.192 to -0.016	0.021
Female (vs. male)	-0.399	-2.925 to 2.126	0.757
Shift work (yes vs. no)	-1.912	-4.213 to 0.389	0.103

Model $R^2 = 0.4474$; n = 290. Two-sided p-values and 95% confidence intervals use HC3 standard errors with an asymptotic standard normal reference distribution.

Coding: female = 1 for women and 0 for men (original GENERO: 2 = female, 1 = male); shift work = 1 for yes and 0 for no; work–private-life interference = SD2 + SD3 + SD4 (each coded 0/1). PSQI uses PSQI_7C_TOTAL. The sensitivity model is exploratory and does not eliminate residual confounding.

Table S4. Exploratory post hoc sensitivity models including concurrent psychological distress (n = 290)

**A. DASS-21 subscales**			
**Predictor**	**B**	**95% CI**	**p**
Intercept	5.1243	0.9559 to 9.2926	0.016
PSQI seven-component global score	0.7609	0.4449 to 1.0769	<0.001
AAQ-II total score	0.0577	-0.0767 to 0.1921	0.4005
Work–private-life interference (0–3)	2.9636	2.0715 to 3.8557	<0.001
Age (years)	-0.0657	-0.1494 to 0.0180	0.1242
Female (vs male)	-0.6488	-2.9196 to 1.6221	0.5755
Shift work (yes vs no)	-1.354	-3.5913 to 0.8832	0.2355
DASS-21 depression (raw)	-0.1617	-0.5872 to 0.2638	0.4563
DASS-21 anxiety (raw)	0.2611	-0.1936 to 0.7159	0.2604
DASS-21 stress (raw)	0.5276	0.0949 to 0.9603	0.0169
Model R^2 = 0.5039			
**B. Aggregated DASS-21 symptoms**			
Predictor	B	95% CI	p
Intercept	5.8515	1.7289 to 9.9742	0.0054
PSQI seven-component global score	0.7665	0.4562 to 1.0768	<0.001
AAQ-II total score	0.0481	-0.0830 to 0.1791	0.4723
Work–private-life interference (0–3)	3.0327	2.1407 to 3.9248	<0.001
Age (years)	-0.0752	-0.1584 to 0.0079	0.0762
Female (vs male)	-0.7129	-3.0126 to 1.5869	0.5435
Shift work (yes vs no)	-1.3754	-3.6054 to 0.8546	0.2267
DASS-21 aggregate (sum of raw subscales)	0.2191	0.1214 to 0.3168	<0.001
Model R^2 = 0.4965			

Notes. Outcome: nine-item MBI-HSS emotional exhaustion sum. Models are exploratory and post hoc. HC3 heteroscedasticity-consistent standard errors, two-sided normal-reference confidence intervals and p-values. Female coded 1 for female, 0 for male; shift work coded 1 for yes, 0 for no. DASS-21 raw subscale scores range from 0 to 21 (not doubled). The aggregated DASS-21 score is the sum of the three raw subscales. These concurrent measures do not establish causal mediation or construct redundancy.

AAQ-II incremental in-sample R^2 in Model A: 0.5039 − 0.5025 = 0.0014. In Model B, incremental R^2 attributable to AAQ-II is approximately 0.0010.

Supplementary File S1. STROBE Cross-Sectional Reporting Checklist — Reporting Cross-Reference

Manuscript: Sleep Quality, Psychological Inflexibility, and Work–Private-Life Interference in Relation to Emotional Exhaustion among Ecuadorian Nursing Assistants: A Cross-Sectional Study

Item	Reporting element (paraphrased)	Manuscript location	Reporting details
1a	Study design in title or abstract	Title; Abstract	Reported
1b	Informative abstract of methods and findings	Abstract	Reported
2	Background and rationale	§1 Introduction	Reported
3	Specific objectives	§1 Introduction, final paragraph	Reported
4	Key elements of study design	§2.1 Study Design and Participants	Reported
5	Setting, locations and relevant dates	§2.1; §2.3	Data collection April–August 2022 reported in §2.3; institution-level recruitment dates not independently verified.
6a	Eligibility criteria, participant sources and selection	§2.1; §2.3	Partially reported: recruitment denominator unavailable
6b	Matching criteria (if applicable)	Not applicable	Not applicable
7	Outcomes, exposures, predictors, confounders and diagnostic criteria	§2.4; §2.5	Reported
8	Data sources and measurement methods	§2.4; File S1.2	Seven-component scoring and 290 calculable totals described in §2.4; File S1.2 referenced.
9	Efforts to address potential bias	§2.5; §4.2	Partially reported
10	Study size rationale	§2.1	Explicitly states no a priori sample-size calculation for this subgroup; recruitment denominator unavailable.
11	Handling of quantitative variables	§2.4; §2.5	Reported
12a	Statistical methods, including confounding control	§2.5	Reported
12b	Methods for subgroups and interactions	§2.5; §3.5–3.6	Sensitivity models reported; no interaction analysis identified
12c	Treatment of missing data	§2.5; §3.2; Table S2	Reported
12d	Sampling strategy in analysis	§2.1; §2.5	Nonprobability sample; no survey weighting identified
12e	Sensitivity analyses	§2.5; §3.4–3.6; File S1	Reported
13a	Participant counts at each study stage	§2.1; §3.1–3.2	Partially reported: recruitment denominators unavailable
13b	Reasons for non-participation	§2.1; §4.2	Partially reported
13c	Participant flow diagram	Not identified	No flow diagram identified; narrative gives N=314 and PSQI n=290, but subgroup invitation denominator unavailable.
14a	Participant characteristics and potential confounders	§3.1; Table 1	Reported
14b	Number of missing data for each variable	§3.2; Table S2; PSQI scoring appendix	PSQI: 290 calculable and 24 non-calculable scores; completeness

			of other variables is not independently established by this cross-reference.
14c	Follow-up time (if applicable)	Not applicable	Not applicable
15	Outcome summary data	§3.2; Table 2	Reported
16a	Unadjusted and adjusted estimates with precision	§3.3–3.6; Tables 3–5; Tables S3–S4	Reported
16b	Category boundaries for categorized variables	§2.4; §3.2	Reported for PSQI
16c	Absolute risk translation (if applicable)	Not applicable	Not applicable
17	Other analyses	3.4–3.6; File S1	Reported
18	Summary of key results	4 Discussion; 5 Conclusions	Reported
19	Study limitations and possible bias	4.2 Limitations	Reported
20	Cautious overall interpretation	4 Discussion; Conclusions	Reported
21	Generalisability	4.2 Limitations	Reported
22	Funding and role of funders	Funding statement	UTPL funding and project code reported

Reporting note: The recruitment denominator for this nursing-assistant subgroup was unavailable, as disclosed in the manuscript. The study approval and data-collection period are reported in the manuscript. Reference: von Elm et al. (2007). The STROBE Statement. PLoS Medicine, 4(10), e296. Checklist reporting elements are paraphrased.

Supplementary File S2. Exploratory regression diagnostics and sensitivity analyses

All calculations were reproduced using the final dataset of 314 Ecuadorian nursing assistants. The analytic sample comprised 290 participants with calculable seven-component PSQI scores. The primary outcome was MBI-HSS emotional exhaustion; predictors were the PSQI seven-component total, AAQ-II total, and the sum of the binary SD2–SD4 work–private-life interference items. These exploratory checks do not replace the primary regression estimates reported in the manuscript.

Table S5. Model diagnostics and functional form

Check	Reproduced result	Interpretation
Primary OLS fit	$R^2 = 0.4318$	290 complete cases
Breusch–Pagan LM test	LM = 8.3637; $p = 0.0391$	Evidence of heteroscedasticity
Cook's distance screen	27/290 above 4/n = 0.01379	Screening flag, not automatic exclusion
Categorical interference model	$R^2 = 0.4341$	Versus 0.4318 with linear 0–3 score
Robust nonlinearity Wald test	$\chi^2(2) = 0.7152$; $p = 0.6993$	No evidence of departure for interference
PSQI quadratic term, HC3 normal reference	B = 0.07170; $p = 0.00176$	Exploratory signal; differs slightly from manuscript $p = 0.0015$
PSQI quadratic term, HC3 t reference	$p = 0.00194$	Reference distribution affects p-value
Interference score excluding SD2	B = 4.6426; $R^2 = 0.4211$	Reduced 2-item indicator; not a validated replacement

File S2.1. Cook's distance sensitivity check

A post hoc deletion sensitivity analysis excluded the 27 observations above the Cook's distance screening threshold, leaving n = 263. This is not recommended as the primary analysis; observations were not identified as erroneous solely because of their influence statistic.

Predictor	Primary model B (n = 290)	After screening exclusion B (n = 263)
PSQI seven-component total	0.9149	0.7307
AAQ-II total	0.1956	0.2500
Work–private-life interference	3.3855	3.2600
Model R^2	0.4318	0.4903

All three estimated predictor associations remained positive after screening exclusion, although their magnitudes changed. The difference does not establish that the excluded cases are invalid. HC3 robust standard errors address heteroscedasticity in coefficient inference but do not establish correct functional form, independence of observations, or absence of institution-level clustering.

The exploratory PSQI quadratic model was reproduced using HC3 robust standard errors and an asymptotic normal reference (n = 290): PSQI-squared B = 0.071699, 95% CI 0.026768–0.116631, $p = 0.001762$ (reported as $p = 0.0018$), $R^2 = 0.448168$. The reduced-indicator confidence interval uses the t reference specified for that separate sensitivity analysis.

Reproduction method: ordinary least squares; HC3 sandwich covariance for robust Wald tests; Breusch–Pagan LM test on the primary OLS residuals; Cook's distance from the primary OLS fit. All tests are exploratory and observational.

File S2.2. PSQI Scoring and Data-Cleaning Appendix

Supplementary documentation of PSQI scoring and data cleaning

1. Instrument and documented scoring framework

The original UTPL project guide describes the Pittsburgh Sleep Quality Index (PSQI) as comprising 19 self-reported items grouped into seven components. Each component is scored from 0 to 3, yielding a global score ranging from 0 to 21. The project guide provides the instrument and its response formats. Participant responses were collected using Survey123, and component and total scores were calculated after data export.

2. Available analytical fields and score checks

The retained analytical dataset includes the seven component fields listed below, the seven-component total (PSQI_7C_TOTAL), and a score-processing status field (PSQI_7C_ESTADO). The following checks were performed on the available analytical fields; they are not a substitute for independent reconstruction from the original deployed Survey123 form.

Component	Analytical field
1	PSQI_C1_CALIDAD_SUBJETIVA
2	PSQI_C2_LATENCIA
3	PSQI_C3_DURACION
4	PSQI_C4_EFICIENCIA
5	PSQI_C5_PERTURBACIONES
6	PSQI_C6_MEDICACION
7	PSQI_C7_DISFUNCION_DIURNA

Among the 314 records, 290 were classified as having a calculable seven-component total. For all 290 records, the seven component scores fell within 0–3, and PSQI_7C_TOTAL equaled the arithmetic sum of those seven components. The seven-component total was the score used for the PSQI-dependent analyses (n = 290).

A separate analytical field named PSQI was also present. In the 290 calculable records, it equaled the sum of six components, omitting component 4 (habitual sleep efficiency). Consequently, PSQI and PSQI_7C_TOTAL differed in 121 records. This is an observed arithmetic relationship between existing analytical fields; it does not establish how the separate field was originally generated.

3. Non-calculable PSQI totals and analytical eligibility

Analytical status label	Number of records
Time in bed recorded as zero	12
Invalid sleep duration	11
Invalid bedtime/wake time	1
Total non-calculable	24

These categories reproduce the processing-status labels recorded in the analytical dataset; they should not be interpreted as independently verified item-level causes from the original deployed Survey123 form. In the 24 non-calculable records, PSQI_7C_TOTAL was blank in the final analytical dataset.

Component-level pattern in the 24 non-calculable records. In the final analytical dataset, components 1, 2, 3, 5, 6, and 7 retained numeric scores within the 0–3 range for all 24 records.

Component 4 (habitual sleep efficiency), PSQI_7C_TOTAL, and PSQI_7C_CATEGORIA were blank. These field-level observations do not independently establish the original item-level causes.

Inclusion in the overall study dataset (N = 314) was distinct from eligibility for PSQI-dependent analyses (n = 290). The latter required a calculable seven-component PSQI total. The available records do not independently establish the operational questionnaire-completion rule used at the point of Survey123 submission.

4. Scope of documented scoring checks

The original UTPL project guide documents the Spanish PSQI questionnaire and its response options. The retained analytical dataset includes original sleep-response fields as well as derived component fields, allowing checks of recorded responses and calculated scores. The verified seven-component arithmetic total and the recorded non-calculable status categories are reported above. These checks do not establish complete independent validation of the original deployed Survey123 configuration, every temporal conversion, or all item-level recoding decisions. No missing scores were imputed.

This appendix reports the checks supported by the available documentation and analytical data; it does not claim an independently reproduced item-level scoring pipeline.
